# Simultaneous Quantitation of Free Amino Acids, Nucleosides and Nucleobases in *Sipunculus nudus* by Ultra-High Performance Liquid Chromatography with Triple Quadrupole Mass Spectrometry

**DOI:** 10.3390/molecules21040408

**Published:** 2016-03-25

**Authors:** Yahui Ge, Yuping Tang, Sheng Guo, Xin Liu, Zhenhua Zhu, Lili Zhang, Pei Liu, Shaoxiong Ding, Xiangzhi Lin, Rurong Lin, Jin-ao Duan

**Affiliations:** 1 Jiangsu Collaborative Innovation Center of Chinese Medicinal Resources Industrialization, and National and Local Collaborative Engineering Center of Chinese Medicinal Resources Industrialization and Formulae Innovative Medicine, Nanjing University of Chinese Medicine, Nanjing 210023, China; geyahui_jsnt@163.com (Y.G.); gsh916@gmail.com (S.G.); liuxin_njutcm@163.com (X.L.); 18913133908@163.com (Z.Z.); lizhang2020@126.com (L.Z.); peiliu1981@126.com (P.L.); dja@njucm.edu.cn (J.D.); 2 College of Ocean and Environment, Xiamen University, Xiamen 361005, China; sxding@xmu.edu.cn; 3 Third Institute of Oceanography, State Oceanic Administration, Xiamen 361005, China; xzlin@tio.org.cn (X.L.); linrulong@yahoo.com (R.L.)

**Keywords:** UPLC-TQ-MS/MS, *Sipunculus nudus*, free amino acid, nucleoside, nucleobase

## Abstract

To evaluate the nutritional and functional value of *Sipunculus nudus*, a rapid, simple and sensitive analytical method was developed using ultra-high performance liquid chromatography coupled with a triple quadrupole mass detection in multiple-reaction monitoring mode for the simultaneous quantitative determination of 25 free amino acids and 16 nucleosides and nucleobases in *S. nudus* within 20 min, which was confirmed to be reproducible and accurate. The limits of detection (LODs) and quantification (LOQs) were between 0.003–0.229 μg/mL and 0.008–0.763 μg/mL for the 41 analytes, respectively. The established method was applied to analyze 19 batches of *S. nudus* samples from four habitats with two different processing methods. The results showed that *S. nudus* contained a variety of free amino acids, nucleosides and nucleobases in sufficient quantity and reasonable proportion. They also demonstrated that the contents of these compounds in different parts of *S. nudus* were significantly discriminating, which were in the order: (highest) coelomic fluid > body wall > intestine (lowest). The method is simple and accurate, and could serve as a technical support for establishing quality control of *S. nudus* and other functional seafoods. Moreover, the research results also laid foundation for further exploitation and development of *S. nudus*.

## 1. Introduction

*Sipunculus nudus*, a *Sipunculid* species, known as sandworm or haichangzi, has a practically global distribution, except for polar waters. They are unsegmented wormlike animals, comprising two sections namely the trunk (main body) and an introvert (extending and contracting neck-like “feeler”) [1]. Called “marine *Cordyceps sinensis*” by local residents, it has crisp, tender, fresh and sweet taste, can nourish internal organs and clear the internal heat [2], and is ranked as a valuable seafood and senior supplement [3,4]. However, since the 1980s, *S. nudus* has suffered from predatory and unhindered exploration stimulated by the market price, combined with more and more serious pollution of oceans which have led to a sharp decrease in the availability of the wild resource and therefore, more and more attention has been paid to artificial cultivation of the worms [5]. As the technology has developed, the amount of *S. nudus* has increased rapidly and how to utilize the resource reasonably has become a major study topic.

In the last decades, *S. nudus* extract was reported to be rich in a variety of nutritional and functional components consisting of free amino acids, fatty acids, polysaccharides, mineral elements and so on. [6,7,8,9]. As we all know, the free amino acids associated with many functional foods such as *Ziziphus jujube*, royal jelly and *Calculus bovis* have received considerable attention [10,11,12]. In recent years, nucleosides and nucleobases have also been proven as important nutritional and functional foodstuffs related to multiple properties such as modulation of the immune response, metabolism, angiocarpy and nervous system as well as antimicrobial and antiviral effects [13]. However, there has been no report about the nucleosides and nucleobases in *S. nudus* so far. Therefore, in order to compile comprehensive information about the nutritional and functional components in *S. nudus*, we performed a preliminary experiment to detect the nucleosides and nucleobases in *S. nudus* and found such constituents were abundant in *S. nudus* water extract.

In the past years, many researches have been carried out on free amino acids, nucleosides and nucleobases as quality control markers of several functional foods such as *Geosaurus*, brown seaweeds, royal jelly, *Ganoderma lucidum* and so on [7,14,15,16,17]. However, there are no definite quality control markers for *S. nudus*, although the free amino acids of *S. nudus* have been detected with low sensitivity by visible spectrophotometry after derivatization and complex pretreatment procedures [18]. There have no reports about the contents and proportion of free amino acids, nucleosides and nucleobases in *S. nudus* till now, making it very necessary to develop a fast, convenient and efficient method to precisely measure the amount of these nutritional constituents in *S. nudus* extract, which will be beneficial for expanding its potential value as well as quality control.

In the traditional way, when *S. nudus* is consumed in dishes [2], the internal parts including the intestine and coelomic fluid are usually removed, and then it is cooked alone or with other food materials. The useful constituents in *S. nudus* may be broken down because of high temperature suing during the processing. Therefore, it is necessary to determine the content variation of free amino acids, nucleosides and nucleobases in different parts of *S. nudus* with different methods of sample preparation for the sake of best developing *S. nudus* as a functional seafood.

Ultra-high performance liquid chromatography (UPLC), coupled with mass spectrometry (MS) detection is an important analytical method that has been developed in recent years [19,20,21]. Due to its efficient separation, high selectivity and high sensitivity, it has been widely used for the quantification and qualitative analysis of active components in biological fluids, medicinal materials and so on [22,23,24,25,26].

In our previous research, the amino acids, nucleosides and nucleobases in another seafood were analyzed by using hydrophilic interaction ultra-performance liquid chromatography coupled with triple quadrupole tandem mass spectrometry [27]. Since the method is simple and accurate, in this study, we also used such a method for simultaneous identification and quantification of 25 free amino acids and 16 nucleosides and nucleobases in different parts of *S. nudus* collected from four habitats with different preparation methods. Then, the data were further handled by a PCA scatter plot to compare the content variation of the samples. The determination of these important components in *S. nudus* could be vital to quality control as well as tapping its full nutritional and functional value.

## 2. Results and Discussion

### 2.1. Sample Preparation Optimization

To identify as many target components as possible in different parts of *S. nudus*, the extraction solvent (water, aqueous methanol of different concentrations), solvent volume (10, 20, 30, 40, 50, and 60 mL), extraction temperature (20, 40, 60, 80, 100 °C), extraction method (refluxing and ultrasonication) and extraction time (10, 20, 30, 40, 50 and 60 min) conditions of the samples from different parts (1.0 g, SE, SI, SC) were optimized. All of parameters were investigated by a univariate method using peak area as a measurement. It was found that the best extraction conditions were ultrasonication at 40 °C for 60 min with 40 mL water as solvent. However, to evaluate the free amino acids, nucleosides and nucleobases of *S. nudus* extracted in the traditional way, the extraction method of refluxing for 60 min was applied to imitate water decoction.

### 2.2. UPLC-TQ-MS/MS Conditions Optimization

In preliminary tests two columns, an Acquity BEH C18 (100 mm × 2.1 mm, 1.7 μm) and an Acquity BEH Amide (100 mm × 2.1 mm, 1.7 μm), were compared to obtain chromatograms with better resolution of adjacent peaks, improved peak shape and shortest peak appearance time. On account of the fact that free amino acids, nucleosides and nucleobases are hydrophilic components with high polarity, the results showed that the latter one had a stronger retention ability as well as better resolution under the same mobile phase and other instrument condition circumstances.

**Figure 1:**
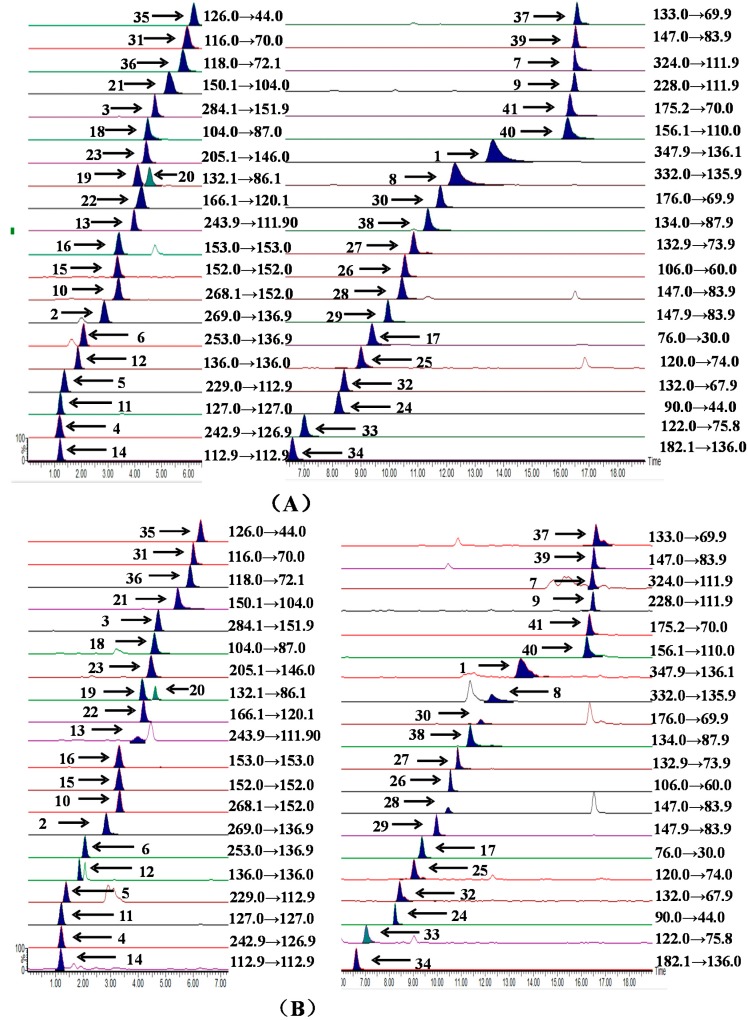
UHPLC–TQ-MS/MS multiple-reaction monitoring (MRM) chromatograms of the mixed standards (**A**) and Sample F1 (**B**). The compound numbers on the chromatograms were adenosine-5′-monophosphate (**1**), inosine (**2**), guanosine (**3**), thymidine (**4**), 2′-deoxyuridine (**5**), 2′-deoxyinosine (**6**), cytidine-5′-monophosphate (**7**), 2′-deoxyadenosine-5′-monophosphate (**8**), 2′-deoxycytidine (**9**), 2′-deoxyguanosine (**10**), thymine (**11**), adenine (**12**), cytidine (**13**), uracil (**14**), guanine (**15**), xanthine (**16**), glycine (**17**), GABA (**18**), leucine (**19**), isoleucine (**20**), methionine (**21**), phenylalanine (**22**), tryptophan (**23**), alanine (**24**), threonine (**25**), serine (**26**), asparagine (**27**), glutamine (**28**), glutamate (**29**), citrulline (**30**), proline (**31**), hydroxyproline (**32**), cysteine (**33**), tyrosine (**34**), taurine (**35**), valine (**36**), ornithine (**37**), aspartate (**38**), lysine (**39**), histidine (**40**) and arginine (**41**), respectively. The peaks of guanine (**15**) and xanthine (**16**) were overlapped or embedded.

As for the mobile phase, as a general rule acetonitrile is known as a polar aprotic solvent with better elution ability, separation selectivity and peak shape compared to methanol, and has been proven to be suitable organic solvent for hydrophilic interaction liquid chromatography with short analysis times. Therefore, a high concentration of acetonitrile was used as organic phase and the concentration was decreased in a gradient. The ammonium acetate and ammonium formate dissolved in acetonitrile are highly volatile, and they can improve the separation of amino acids, nucleosides and nucleobases in the UPLC analysis process [28]. Different mobile phases including independent solutions with different concentration and mixed solutions with different concentration of components were compared. The results showed that a mixed solution containing ammonium formate and ammonium acetate as mobile phase salt additives could increase the sensitivity and improve the peak shapes for these components. The retention times and peak shapes of the compounds were influenced by the different concentrations of ammonium formate and ammonium acetate, consequently, 5 mmol/L ammonium formate and ammonium acetate in the organic phase and 1 mmol/L ammonium formate and ammonium acetate in the aqueous phase produced the best shaped peaks in the shortest time. Meanwhile, formic acid is also used to inhibit solute ionization to improve the peak shape so different concentrations of formic acid were added and compared. Eventually, it was determined that the mobile phase should be composed of A (5 mmol/L ammonium formate, 5 mmol/L ammonium acetate and 0.2% formic acid in aqueous solution) and B (1 mmol/L ammonium formate, 1 mmol/L ammonium acetate and 0.2% formic acid in acetonitrile) with gradient elution. As regard to flow rate and column temperature, the ranges were both optimized, and the results show that the best mobile phase flow rate was 0.4 mL/min and the column temperature was maintained at 35 °C. Analytical chromatograms of the mixed standards and Sample F1 are presented in Figure 1.

For the best MS/MS condition of each analyte, all of the compounds were examined separately in direct infusion mode by a full-scan MS method in both positive and negative ionization modes. The results show that both higher sensitivity and clearer mass spectra were obtained in the positive ion mode compared to the negative ion mode. The free amino acids, nucleosides and nucleobases could combine with H^+^ to give [M + H]^+^ quasi-molecular ions in ESI^+^ mode. MRM mode was applied in the experiment, and the influence of nucleosides could be minimized because the peak could appear only when the parent and daughter ions were both detected by choosing the appropriate parent and daughter ions. To obtain the best ion pairs, at least two precursor/product ion pairs were chosen for each analyte for quantitative research and the most sensitive and specific ion pairs were selected for the MRM determination. For the nucleosides, [M + H]^+^ were selected as parent ions and [M + H − deoxyribose]^+^ and [M + H − ribose]^+^ was selected as daughter ions . As for nucleobases, we have tried to split these compounds before, however, the abundance of product ions for these compounds is too low to be detected by MRM. Consequently, we chose [M + H]^+^ as both parent and daughter ions for these compounds. For most α-amino acids, [M + H − HCOOH]^+^ were rearranged to [R − CH = NH_2_]^+^ as daughter ions. For γ-aminobutyric acid, [M + H − NH_3_]^+^ was rearranged to [R − CH − COOH]^+^ as daughter ion.

For glutamine and asparagine, [M + H − HCOOH − NH_3_]^+^ were selected as daughter ions. For alkaline amino acids such as arginine, lysine, citrulline and so on, their daughter ions could be affected by the presence of the amino groups of every amino acid (Table 1). Then we optimized cone voltage and collision energy by the function of Intellistart software in the Waters XevoTM TQ MS system.

### 2.3. Method Validation

The established chromatographic method was validated by determining the linearity, LOD, LOQ, intra- and inter-day precisions, repeatability, stability, and recovery. The correlation coefficient values (*r*^2^) showed all calibration curves exhibited good linear regressions (*r*^2^ > 0.9919) within the determination range of the 41 investigated compounds. The LODs (S/N = 3) and LOQs (S/N = 10) of the 41 compounds ranged from 0.003–0.229 μg/mL and 0.008–0.763 μg/mL, respectively. The intraday precisions were investigated by determining analytes in six replicates at known concentration during a single day while the interday precisions were determined during three successive day. And the RSDs serve as the measure of precisions. The results showed that RSD values for the intraday precisions were <3.72%, and for the interday precisions were <3.42%. The repeatability was evaluated by analyzing six samples processed by the same methods, and the RSDs of the repeatability were <4.48%. The storage stability of the sample was measured by analyzing the same sample at 0, 2, 4, 8, 12, 24 h within one day, and the RSDs of the storage stability were <4.92%. The recovery was performed by adding known amount of individual standards into an accurately weighed sample, and the mixture was processed and analyzed by the same methods of the samples. The recoveries were in the range of 94.03% and 106.33% for the 41 compounds and the RSDs were <3.76%. Besides, no significant matrix effects were noted in relatively complex functional food matrices within 24 h. All of above indicated that the established method was accurate enough for the determination of the 41 amino acids, nucleosides and nucleobases in *S. nudus* (Table 2). We have compared the determination results with and without internal standards in our preliminary experiments. The results showed that the optimized conditions of sample preparation, chromatogram and mass spectrum were stable and the contents of the components tested in the samples were quite identical, besides there was little difference between the errors of the methodology both with and without internal standards, so consequently, we decided not to use internal standards by reference to relevant methods in the field [29,30].

### 2.4. Sample Analysis

**Figure 2:**
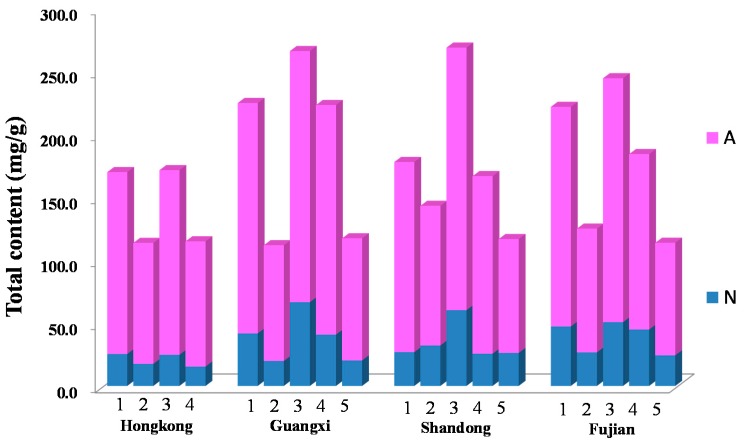
The total contents of 41 compounds in different samples. The numbers on the stacked bar chart indicate body wall, ultrasonication (**1**), intestine, ultrasonication (**2**), coelomic fluid, ultrasonication (**3**), body wall, refluxing (**4**) and intestine, refluxing (**5**). The color A stands for total contents of amino acids, and the color N stands for total contents of nucleosides and nucleobases.

The method was applied to analyze 25 free amino acids and 16 nucleosides and nucleobases in different parts of *S. nudus* collected from four different habitats in China with different sample preparation methods. The results demonstrated that all the samples were rich in these 41 compounds, and the total contents of these investigated compounds varied from 111.70 mg/g to 268.55 mg/g. From the data, it was found that total contents of free amino acids, nucleosides and nucleobases in *S. nudus* extract processed by both ultrasonication and refluxing were pretty much the same or just a little decreased during by the refluxing procedure, the details of which are shown in Table 3. However, the total contents of three parts in *S. nudus* were different, and in the order: (highest) coelomic fluid > body wall > intestine (lowest). As for the habitats, the results showed that *S. nudus* collected from Hong Kong was low in nutritional value compared to the other habitats, while the qualities of the other three habitats are not too different from each other. From the perspective of shape and appearance, it was also proved that the worms inhabiting the waters surrounding Hong Kong are thinner, shorter, have a darker color and contain more silt (Figure 2).

For specific compounds determined in the experiments, remarkable differences were also observed. The contents of free amino acids except GABA were of milligram magnitude, while the contents of nucleosides or nucleobases were mostly of microgram magnitude. The contents of nucleosides or nucleobases were in the following order: nucleobases > deoxynucleosides > ribonucleosides. The concentrations of 2′-deoxyguanosine and its corresponding base were greater than any other nucleosides or nucleobases in all the parts.

2′-Deoxyguanosine is recognized as an oxidative damage biomarker of DNA [31]. The high contents of 2′-deoxyguanosine in the worms may be a result of physical and chemical factors such as foreign chemicals or ionizing radiation besides body metabolism. Additionally, the content of xanthine, reported to be bronchodilator, is of milligram magnitude and second to 2′-deoxyguanosine, which is extremely interesting and worthy of further study. As for free amino acids, *S. nudus* contains all 20 proteinogenic amino acids and five non-protein amino acids. The percentage of eight essential amino acids in the 20 proteinogenic amino acids varied from 23.91% to 33.11%, and the percentage in body wall and intestine was higher than in coelomic fluid. The total contents of five non-protein amino acids in the samples, including GABA, citrulline, hydroxyproline, ornithine and taurine, varied from 7.77 mg/g to 22.90 mg/g, among which the taurine had the highest percentage which varied from 84.60% to 94.60%. Taurine has been reported to improve aerobic endurance, mental performance, concentration and memory [32], and we suggest that it may play an active role in the reported learning and memory enhancing pharmacological action as well as the anti-fatigue and anti-anoxia action of *S. nudus* [33,34]. Notably, the contents of fresh flavor amino acids including glycine, alanine, glutamate, aspartate and so on were fairly high, especially the contents of glycine which were the highest of all the free amino acids in *S. nudus* according to the assays, and all of these fresh flavor amino acids above fully explain its reputed good taste (Table 3).

**Figure 3:**
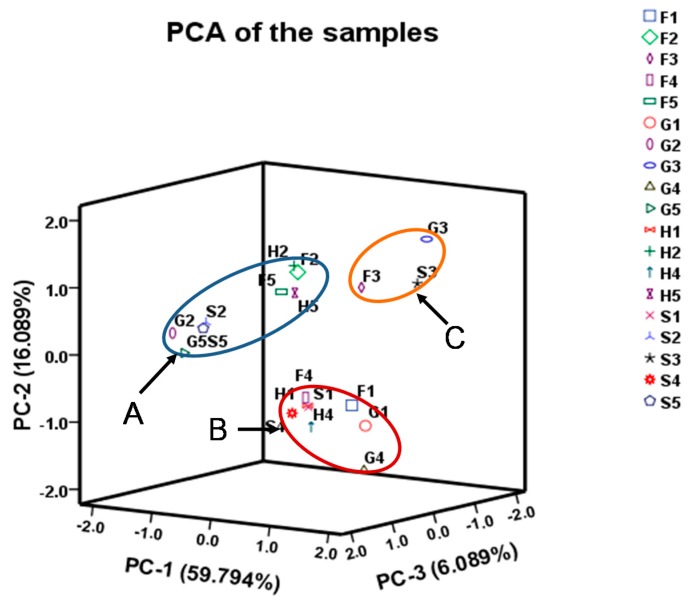
The PCA of the samples. The samples were body wall, ultrasonication (**1**), intestine, ultrasonication (**2**), coelomic fluid, ultrasonication (**3**), body wal, refluxing (**4**) and intestine refluxing (**5**) collected from Fujian (**F**), Guangxi (**G**), Hong Kong (**H**) and Shandong (**S**).

To evaluate the variation of samples, PCA was performed on the basis of the contents of 41 tested compounds from UPLC-TQ-MS profiles. The first three principal components (PC 1, PC 2 and PC 3) with > 83.70% of the whole variance were extracted. Among them, PC 1, PC 2 and PC 3 accounted for 60.63, 17.16 and 5.91% of total variance, respectively. The remaining principal components, which had a minor effect on the model, were discarded. According to their loadings, PC 1 had good correlations with 2′-deoxyinosine, 2′-deoxyguanosine, guanine, xanthine, glycine, isoleucine, threonine, glutamate, proline and histidine, among which the correlation of glycine, threonine and glutamate are over 95%. And PC 2 had good correlation with analytes of 2′-deoxyadenosine-5′-monophosphate and citrulline whereas PC 3 had good correlation with analytes of inosine and taurine (Table 4). In the scatter plot, each sample is represented as a marker. It was noticeable that the samples were clearly clustered into three groups: cluster A (sample H2, F2, G2, S2, H5, F5, G5, S5), cluster B (H1, F1, G1, S1, H4, F4, G4, S4) and cluster C (F3, G3, S3) (Figure 3). This result indicated there are significant differences in the proportions and quantity of the 41 compounds in different parts in *S. nudus* while the impact of the extraction procedure method used was not significant.

## 3. Materials and Methods

### 3.1. Reagents and Materials

**Figure 4:**
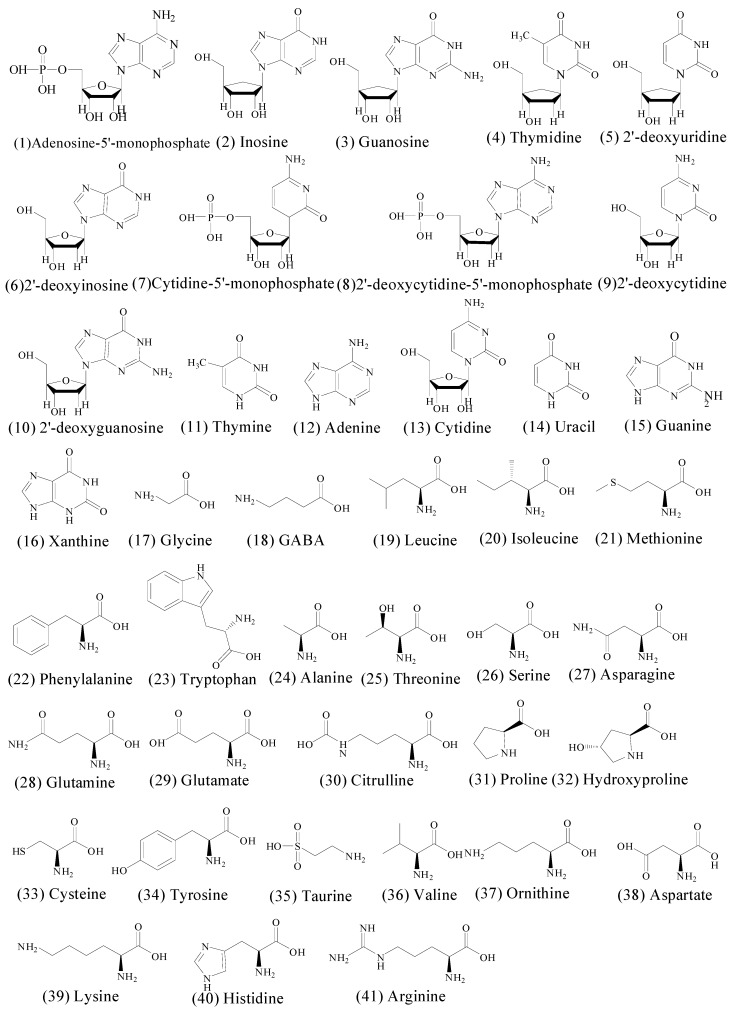
Chemical structures of the 41 identified amino acids nucleosides and nucleobases.

Ammonium formate (Shanghai Lingfeng Chemical Reagent Co., Ltd., Shanghai, China), ammonium acetate (Sinopharm Chemical Reagent Co., Ltd., Shanghai, China), formic acid (Merck Millipore, Darmstadt, Germany) and acetonitrile (Tedia China, Shanghai, China) were analytical grade. Deionized water (H_2_O) was purified by a superpurification system (EPED Technology Development, Nanjing, China). Forty one standards including adenosine-5′-mono-phosphate (**1**), inosine (**2**), guanosine (**3**), thymidine (**4**), 2′-deoxyuridine (**5**), 2′-deoxyinosine (**6**), cytidine-5′-monophosphate (**7**), 2′-deoxyadenosine-5′-monophosphate (**8**), 2′-deoxycytidine (**9**), 2′-deoxyguanosine (**10**), thymine (**11**), adenine (**12**), cytidine (**13**), uracil (**14**), guanine (**15**), glycine (**17**), GABA (**18**), asparagine (**27**), glutamine (**28**), citrulline (**30**), hydroxyproline (**32**), taurine (**35**) and ornithine (**37**) were purchased from Sigma (St. Louis, MO, USA). Standards including xanthine (**16**), leucine (**19**), isoleucine (**20**), phenylalanine (**22**), tryptophan (**23**), alanine (**24**), threonine (**25**), serine (**26**), glutamate (**29**), proline (**31**), tyrosine (**34**), valine (**36**), aspartate (**38**), lysine (**39**), histidine (**40**) and arginine (**41**) were obtained from the National Institute for the Control of Pharmaceutical and Biological Products (Beijing, China). A chemical standard of cysteine (**33**) was obtained from Aladdin Chemical (Shanghai, China). A reference of methionine (**21**) was purchased from Sinopharm Chemical Reagent (Beijing, China). The purity of each compound was > 98%, as determined by UPLC analysis (Figure 4).

A mixed standard stock solution containing the reference compounds 1–41 dried to constant weight was prepared in methanol/water (9:1, *v*/*v*). Working standard solutions for calibration curves were prepared by diluting the mixed standard stock solution with 10% methanol at different concentrations.

### 3.2. Sample Preparation

The samples included three different parts (body wall, intestine, coelomic fluid) of *S. nudus* collected from four different habitats in China in August, 2014 (Table 5). 

**Figure 5:**
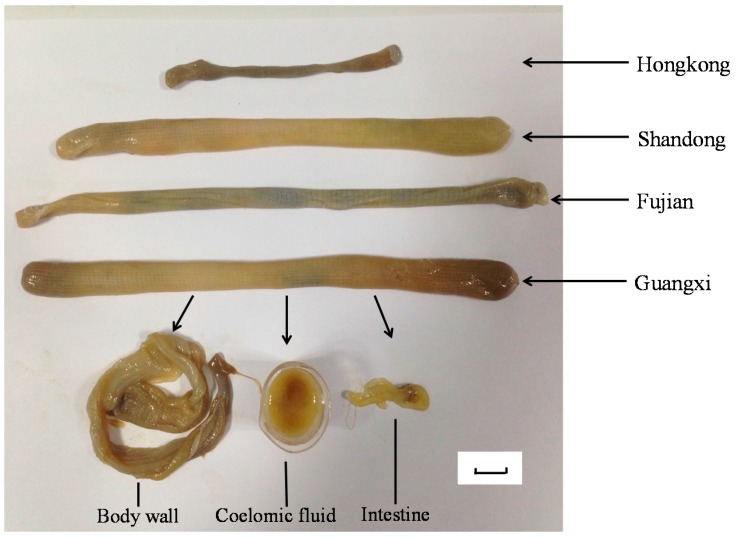
The appearance and anatomy of samples collected from four different habitats.

The *S. nudus* samples collected from the different habitats were identified by Prof. Ding Shaoxiong (Xiamen University, Fujian Province, China). After collection, the animals were kept in a −80 °C refrigerator. Voucher specimens were deposited in Nanjing University of Chinese Medicine, China. After defrosting at the temperature of 4 °C and removing the excreta, the *S. nudus* were dissected into three parts, including body wall (SE), intestine (SI), coelomic fluid (SC) (Figure 5).

All three parts were freeze–dried in a vacuum freeze drier system (Labconco, Kansas City, MO, USA), then weighed and smashed, respectively. One gram of each dry sample was accurately weighed into a 50 mL conical flask, then 40 mL distilled water was added. All of the mixtures were placed into an ultrasonic bath (40 kHz) for 60 min at room temperature. Meanwhile, the same samples were refluxed for 60 min. Water was added to both sets oif samples to compensate for any lost during extraction. After centrifugation (1,3000 r/min) for 10 min, the protein in the supernatants was removed by adding acetonitrile to double the volume and then stored at 4 °C and filtered through 0.22 μm cellulose membrane filters prior to injection.

### 3.3. UPLC-TQ-MS/MS Conditions

The UPLC analysis was performed on a Waters ACQUITY UPLC H-class system (Waters, Milford, MA, USA) equipped with a quaternary pump solvent management system, an on-line degasser and an autosampler. The separation was performed on an ACQUITY UPLC BEH amide column (2.1 mm × 100 mm, 1.7 μm). The mobile phase was composed of A (5 mmol/L ammonium formate, 5 mmol/L ammonium acetate and 0.2% formic acid in aqueous solution) and B (1 mmol/L ammonium formate, 1 mmol/L ammonium acetate and 0.2% formic acid in acetonitrile) with a gradient elution: 0–3 min, 10% A; 3–9 min, 10%–18% A; 9–15 min, 18%–20% A, 15–16 min, 20%–46% A, 16–18 min, 46% A. The flow rate of the mobile phase was 0.4 mL/min, the injection volume was 2 μL, and the column temperature was maintained at 35 °C.

The mass spectrometry detection was performed by using a Xevo^TM^ Triple Quadrupole MS (Waters Corp.) equipped with an electrospray ionisation (ESI) source operating in positive ionisation mode. The desolvation gas flow rate was set to 1000 L/h at a temperature of 350 °C, the cone gas flow rate was set at 20 L/h and the source temperature was set at 120 °C. The capillary voltage was set to 3000 V, the cone voltage and collision energy were set depending upon the MRM for each compound. Data were collected in MRM mode by screening parent and daughter ions simultaneously (Table 1). The raw data were acquired and processed with the Waters MassLynx 4.1 software (Waters China, Shanghai, China).

### 3.4. Data Processing

The raw data were processed by the MassLynx 4.1 software, and the identification of free amino acids, nucleosides and nucleobases was carried out by comparing the retention time of target peaks with those of the standards, and the quantification was calculated by linear calibration plots of the peak. The statistical analysis was performed using the SPSS 17.0 software (SPSS, Chicago, IL, USA).

### 3.5. Method Validation

The linearity was verified by plotting the peak areas *versus* the corresponding concentrations of each analyte. The limit of detection (LOD) and limit of quantification (LOQ) were obtained by diluting the mixed standard working solution to the appropriate concentrations until the S/N for each compound was about 3 and 10, respectively. The intra- and inter-day precisions were examined by analyzing the mixed standard solutions six times in a day and repeatedly in three consecutive days. To confirm the repeatability, six sample solutions from the same sample were prepared and analyzed in parallel. To evaluate the stability of the components, one of the sample solutions was analyzed in various periods (0, 2, 4, 6, 12, and 24 h). Besides, there was also a recovery test was used to evaluate the accuracy of this method. It was performed by adding corresponding marker compounds with high (120%), medium (100%), and low (80%) levels into accurately weighed samples, and then they were processed and analyzed with the same methods above. We prepared two groups of samples which were the standard solution and the matrix matching standard solution adding appropriate amounts of standards to the samples. Then we used the radios (the peak area of matrix matching standards/the peak area of standards) to investigate the matrix effects.

## 4. Conclusions

In this study, a simple, rapid and reliable UPLC-TQ-MS/MS method was developed and applied to simultaneously determine 25 free amino acids and 16 nucleosides and nucleobases in *S. nudus* extracts within 20 min. The method had acceptable intra- and interday precision (RSD < 3.72%, RSD < 3.42%). The LODs and LOQs ranged from 0.003–0.229 μg/mL and 0.008–0.763 μg/mL, respectively. Recoveries were between 94.03% and 106.33% with RSDs in the range of 0.64%–3.76% for all target compounds. Real sample data demonstrate that *S. nudus* is an excellent source of free amino acids, nucleosides and nucleobases with great nutritional and functional value. The contents of these compounds in different parts were significantly different, and in the order: (highest) coelomic fluid > body wall > intestine (lowest). As the contents of xanthine, 2′-deoxyguanosine, taurine and glycine were the highest in each category, all of them could be proposed as markers for quality control of *S. nudus*. Moreover, the research results also provide a firm basis for further exploitation and development of *S. nudus*.

## Figures and Tables

**Table 1:** Precursor/product ion pairs and parameters for MRM of compounds used in this study.

NO	Analytes	MW	RT (min)	MRM Transitions	Cone Voltage (V)	Collision Energy (eV)
1	Adenosine-5′-monophosphate	391	13.75	347.9→136.1	20	20
2	Inosine	268	2.96	269.0→136.9	10	14
3	Guanosine	283	4.88	284.1→151.9	14	14
4	Thymidine	242	1.34	242.9→126.9	10	10
5	2′-Deoxyuridine	228	1.46	229.0→112.9	8	10
6	2′-Deoxyinosine	252	2.19	253.0→136.9	22	12
7	Cytidine-5′-monophosphate	323	16.72	324.0→111.9	16	14
8	2′-Deoxyadenosine-5′-mono-phosphate	329	12.53	332.0→135.9	20	16
9	2′-Deoxycytidine	227	16.65	228.0→111.9	28	10
10	2′-Deoxyguanosine	267	3.55	268.1→152.0	10	12
11	Thymine	126	1.45	127.0→127.0	30	15
12	Adenine	135	2.17	136.0→136.0	30	20
13	Cytidine	243	4.17	243.9→111.9	28	10
14	Uracil	112	1.20	112.9→112.9	30	15
15	Guanine	151	3.58	152.0→152.0	30	20
16	Xanthine	152	3.60	153.0→153.0	30	20
17	Glycine	75	9.53	76.0→30.0	12	6
18	GABA	103	4.86	104.0→87.0	16	10
19	Leucine	131	3.93	132.1→86.1	16	10
20	Isoleucine	131	4.75	132.1→86.1	16	10
21	Methionine	149	5.25	150.1→104.0	14	10
22	Phenylalanine	165	4.32	166.1→120.1	18	14
23	Tryptophan	204	4.80	205.1→146.0	16	18
24	Alanine	89	8.46	90.0→44.0	16	10
25	Threonine	119	9.10	120.0→74.0	38	20
26	Serine	105	10.65	106.0→60.0	14	8
27	Asparagine	132	10.81	132.9→73.9	12	14
28	Glutamine	146	10.60	147.0→83.9	8	16
29	Glutamate	147	10.11	147.9→83.9	12	14
30	Citrulline	175	11.98	176.0→69.9	16	20
31	Proline	131	5.95	116.0→70.0	20	10
32	Hydroxyproline	131	8.61	132.0→67.9	18	16
33	Cysteine	121	7.15	122.0→75.8	14	17
34	Tyrosine	181	6.74	182.1→136.0	16	16
35	Taurine	125	6.27	126.0→44.0	24	14
36	Valine	117	5.65	118.0→72.1	12	10
37	Ornithine	132	17.01	133.0→69.9	14	14
38	Aspartate	133	11.68	132.9→73.9	12	14
39	Lysine	146	16.77	147.0→83.9	14	14
40	Histidine	155	16.53	156.1→110.0	22	14
41	Arginine	174	16.68	175.2→70.0	22	18

The peaks of guanine (**15**) and xanthine (**16**) were overlapped.

**Table 2:** Calibration curves, LOD, LOQ, precision, repeatability, stability and recovery of the 41 analytes.

Analyte ^a^	Calibration Curve	*r^2^*	Linear Range (μg/mL)	LOD (μg/mL)	LOQ (μg/mL)	Precision RSD, %, *n* = 6		Repeatablity (RSD%) (*n* = 6)	Stability (RSD%) (*n* = 6)	Recovery (%, *n* = 3)		Matrix Effect ^b^
						Intraday	Interday			Mean	RSD	
1	y = 3470.2X − 4115.1	0.9975	0.57–28.25	0.023	0.077	1.34	0.89	3.05	3.71	99.72	1.91	0.95
2	y = 17,200.4X + 4024.2	0.9982	0.15–7.69	0.021	0.069	1.02	1.03	3.03	4.92	99.86	2.87	0.92
3	y = 12,693.4X + 481.18	0.9998	0.24–11.75	0.007	0.025	0.75	0.39	2.57	2.27	105.13	3.47	1.02
4	y = 5675.6X − 847.53	0.9951	1.17–58.50	0.033	0.110	2.84	2.92	3.12	4.90	98.65	1.11	0.93
5	y = 651.92X + 50.414	0.9996	0.51–25.25	0.080	0.267	2.65	2.90	2.38	4.66	94.64	1.54	0.98
6	y = 8691.2X − 303.63	0.9999	1.09–54.50	0.015	0.050	2.03	2.22	3.03	3.43	98.56	2.54	0.92
7	y = 5984.3X − 5771.5	0.9997	0.61–30.25	0.032	0.106	1.42	1.05	1.73	2.15	102.73	2.01	0.94
8	y = 13,359.8X − 12,482	0.9992	0.49–24.50	0.083	0.278	1.43	0.70	3.11	2.17	104.49	2.41	0.96
9	y = 31,588X − 42,789	0.9928	0.29–14.50	0.003	0.008	2.98	3.02	2.55	1.36	99.59	3.14	1.01
10	y = 11,200X − 9094.5	0.9961	0.86–43.13	0.012	0.041	3.72	2.74	2.12	3.31	101.85	2.86	0.97
11	y = 10,862.6X + 510.08	0.9999	1.60–79.69	0.078	0.261	0.99	1.03	3.71	2.42	98.43	1.13	0.94
12	y = 83,008X + 23,366	0.9923	0.11–5.37	0.040	0.131	1.04	1.16	2.73	2.90	101.53	3.76	0.93
13	y = 338.72X − 22.948	0.9985	0.29–14.25	0.074	0.247	0.88	0.98	1.51	3.60	98.01	1.76	1.03
14	y = 1246.64X − 83.519	0.9999	0.56–27.75	0.204	0.681	1.39	1.98	2.43	3.12	99.76	2.03	0.98
15	y = 3587.4X + 351.61	0.9995	1.19–59.38	0.021	0.071	3.10	3.42	2.76	3.39	100.04	2.86	0.91
16	y = 396.14X − 47.85	0.9969	0.35–17.66	0.229	0.763	1.52	1.53	3.01	2.55	102.24	1.78	0.93
17	y = 457X + 281.7	0.9973	1.80–90.00	0.204	0.683	0.98	0.15	2.78	2.91	102.08	2.18	0.95
18	y = 66,192X + 262.14	0.9919	0.29–14.44	0.031	0.105	0.25	0.29	2.33	3.88	106.69	0.64	0.97
19	y = 66,398X + 140,788	0.9925	1.78–89.07	0.018	0.059	0.45	0.51	2.53	3.95	96.86	3.07	0.92
20	y = 33,258X + 18,095	0.9922	0.31–15.23	0.031	0.105	0.76	0.77	1.72	4.23	104.21	3.08	0.96
21	y = 11,852.6X − 1761.7	0.9997	0.91–45.70	0.016	0.055	1.14	0.93	2.70	3.11	101.27	2.36	0.97
22	y = 29,260X + 15,598	0.9968	0.34–16.88	0.023	0.076	1.93	2.13	2.13	2.10	99.63	1.53	0.92
23	y = 10,330.4X + 358.31	0.9999	1.95–97.66	0.040	0.132	0.57	0.58	1.36	4.33	100.78	3.09	0.93
24	y = 6520.8X + 1836.6	0.9999	0.57–28.28	0.060	0.200	1.39	1.37	1.44	3.86	99.45	2.60	0.93
25	y = 103.41X + 374.97	0.9940	0.35–17.70	0.098	0.325	1.75	2.01	3.33	3.31	104.37	1.47	1.02
26	y = 2565.1X + 1383.7	0.9992	0.34–16.88	0.037	0.121	0.95	1.00	1.19	2.94	95.94	2.51	0.99
27	y = 8145.2X − 3078	0.9985	0.22–10.98	0.023	0.075	0.60	0.56	3.29	2.77	100.14	3.17	0.92
28	y = 4690.1X + 1299.4	0.9995	0.22–11.21	0.029	0.096	0.55	0.62	2.93	3.64	97.65	2.92	0.94
29	y = 8301.8X − 1385.6	0.9994	6.94–346.88	0.060	0.202	0.84	0.91	1.98	4.85	94.03	3.33	0.96
30	y = 5115.8X + 1696	0.9995	0.51–25.59	0.028	0.092	0.86	0.67	0.88	2.67	106.07	2.09	1.01
31	y = 87,432X + 28019	0.9966	1.92–96.09	0.031	0.105	1.45	1.48	2.35	3.05	96.84	3.93	0.95
32	y = 2526.8X − 1496.4	0.9968	0.34–16.80	0.026	0.086	0.99	1.07	1.03	2.58	98.26	2.16	0.95
33	y = 1985.54X − 1440	0.9999	0.07–3.42	0.074	0.246	0.83	0.67	1.78	2.56	99.55	2.74	1.03
34	y = 5755.6X − 3405.1	0.9994	1.64–82.03	0.028	0.094	1.02	0.96	1.90	3.34	100.67	2.72	0.97
35	y = 1091.9X + 198.76	0.9986	0.60–30.16	0.048	0.161	1.07	1.23	2.21	3.36	103.23	1.74	0.92
36	y = 42,856X − 4351.9	0.9999	1.82–91.02	0.027	0.090	1.20	0.99	1.37	2.47	95.06	2.67	0.94
37	y = 8726.2X − 7910.9	0.9986	0.89–44.53	0.051	0.170	1.68	1.44	0.98	1.02	103.28	2.38	0.91
38	y = 3054.6X + 100.91	0.9999	3.75–187.50	0.106	0.352	1.45	1.68	1.90	3.04	105.55	1.85	0.98
39	y = 10,841.6X − 6708.3	0.9996	7.81–390.63	0.050	0.168	1.23	0.96	4.48	2.46	102.56	2.83	0.97
40	y = 12,365.8X + 2691.9	0.9990	1.83–91.41	0.018	0.060	1.51	0.55	3.15	2.88	100.41	3.07	0.95
41	y = 11,109.2X + 30,682	0.9998	0.60–30.16	0.024	0.080	2.08	1.42	2.57	1.35	106.33	1.50	0.91

^a^ Analyte number. ^b^ Matrix effects are calculated by slope matrix/slope solvent.

**Table 3:** Contents (mg/g) of amino acids, nucleosides and nucleobases in *Sipunculus nudus*.

**Analyte ^a^**	**Samples (mg/g, *n* = 3)**									
	**H1**	**G1**	**S1**	**F1**	**H2**	**G2**	**S2**	**F2**	**G3**	**S3**
1	0.15 ± 0.02	0.15 ± 0.02	0.18 ± 0.02	0.18 ± 0.01	0.18 ± 0.03	0.18 ± 0.00	0.21 ± 0.01	0.18 ± 0.01	0.27 ± 0.03	0.25 ± 0.04
2	0.03 ± 0.01	0.01 ± 0.00	0.06 ± 0.01	0.04 ± 0.01	0.19 ± 0.02	0.10 ± 0.02	0.04 ± 0.02	0.04 ± 0.00	0.12 ± 0.02	0.05 ± 0.01
3	0.14 ± 0.04	0.05 ± 0.01	0.09 ± 0.01	0.06 ± 0.02	0.08 ± 0.01	0.33 ± 0.04	0.13 ± 0.01	0.08 ± 0.02	0.21 ± 0.02	0.10 ± 0.01
4	0.90 ± 0.09	1.30 ± 0.28	0.97 ± 0.06	1.78 ± 1.21	0.73 ± 0.12	0.63 ± 0.09	1.11 ± 0.39	0.98 ± 0.05	2.84 ± 0.96	3.22 ± 0.24
5	0.16 ± 0.01	0.70 ± 0.02	0.38 ± 0.03	0.75 ± 0.06	0.16 ± 0.02	0.18 ± 0.00	0.45 ± 0.04	0.53 ± 0.06	1.22 ± 0.22	1.18 ± 0.24
6	0.70 ± 0.08	1.24 ± 0.21	0.83 ± 0.03	1.80 ± 0.51	0.41 ± 0.02	0.44 ± 0.03	0.89 ± 0.04	0.64 ± 0.02	2.66 ± 0.18	2.86 ± 0.16
7	0.16 ± 0.01	0.18 ± 0.01	0.19 ± 0.02	0.18 ± 0.00	0.16 ± 0.02	0.17 ± 0.01	0.19 ± 0.01	0.16 ± 0.02	0.31 ± 0.02	0.27 ± 0.04
8	0.11 ± 0.02	0.15 ± 0.01	0.13 ± 0.00	0.16 ± 0.02	0.24 ± 0.04	0.17 ± 0.01	0.26 ± 0.04	0.27 ± 0.04	0.35 ± 0.05	0.36 ± 0.06
9	0.55 ± 0.17	0.21 ± 0.14	0.41 ± 0.08	0.30 ± 0.03	0.43 ± 0.01	0.43 ± 0.02	0.28 ± 0.03	0.17 ± 0.02	0.34 ± 0.04	0.41 ± 0.03
10	12.34 ± 1.31	21.45 ± 0.68	11.50 ± 0.47	25.07 ± 1.68	6.63 ± 0.86	7.57 ± 0.61	15.41 ± 1.13	11.61 ± 0.42	31.53 ± 3.17	28.66 ± 2.16
11	1.19 ± 0.31	1.52 ± 0.22	1.24 ± 0.41	2.17 ± 0.26	0.94 ± 0.16	0.87 ± 0.13	1.33 ± 0.06	1.19 ± 0.28	3.15 ± 0.04	3.51 ± 0.65
12	0.08 ± 0.01	0.18 ± 0.01	0.12 ± 0.02	0.14 ± 0.01	0.07 ± 0.00	0.06 ± 0.01	0.12 ± 0.03	0.14 ± 0.00	0.28 ± 0.01	0.22 ± 0.02
13	0.10 ± 0.02	0.10 ± 0.01	0.12 ± 0.01	0.09 ± 0.01	0.07 ± 0.01	0.55 ± 0.01	0.85 ± 0.12	0.09 ± 0.00	0.07 ± 0.01	0.19 ± 0.01
14	0.18 ± 0.04	0.62 ± 0.08	0.37 ± 0.01	0.62 ± 0.03	0.17 ± 0.01	0.11 ± 0.00	0.40 ± 0.02	0.37 ± 0.03	1.25 ± 0.16	1.40 ± 0.03
15	1.60 ± 0.34	2.48 ± 0.64	1.85 ± 0.67	2.59 ± 0.74	1.27 ± 0.17	1.49 ± 0.37	1.92 ± 0.34	1.89 ± 0.24	4.08 ± 0.07	3.17 ± 0.35
16	7.01 ± 0.89	11.39 ± 1.68	8.44 ± 0.53	11.36 ± 1.23	5.84 ± 0.75	6.60 ± 0.61	8.61 ± 0.62	8.39 ± 0.32	17.91 ± 1.21	14.36 ± 0.81
17	26.84 ± 2.14	50.01 ± 4.95	30.62 ± 2.87	45.00 ± 4.61	18.07 ± 1.14	12.79 ± 0.81	25.86 ± 2.27	26.42 ± 2.12	60.59 ± 4.65	63.16 ± 5.21
18	0.07 ± 0.01	0.06 ± 0.00	0.05 ± 0.01	0.05 ± 0.02	0.03 ± 0.00	0.03 ± 0.01	0.03 ± 0.00	0.05 ± 0.01	0.06 ± 0.00	0.05 ± 0.02
19	5.03 ± 0.87	5.43 ± 1.01	4.95 ± 0.21	5.70 ± 0.28	2.64 ± 0.75	3.89 ± 0.21	3.68 ± 0.35	2.82 ± 0.76	5.21 ± 1.01	5.29 ± 0.78
20	3.13 ± 0.33	4.27 ± 0.36	3.50 ± 1.08	4.19 ± 0.51	1.48 ± 0.26	2.57 ± 0.13	2.25 ± 0.12	1.86 ± 0.73	4.39 ± 0.64	4.55 ± 0.17
21	0.85 ± 0.02	0.91 ± 0.01	0.81 ± 0.02	0.88 ± 0.01	1.36 ± 0.48	0.52 ± 0.05	0.48 ± 0.09	0.37 ± 0.03	0.76 ± 0.01	0.84 ± 0.16
22	5.21 ± 1.18	6.19 ± 1.22	5.60 ± 0.52	6.47 ± 0.79	2.70 ± 1.03	4.39 ± 0.23	5.00 ± 1.24	3.59 ± 0.12	5.62 ± 0.46	5.81 ± 0.74
23	0.93 ± 0.03	1.60 ± 0.06	1.14 ± 0.03	1.52 ± 0.04	0.55 ± 0.05	0.86 ± 0.04	1.09 ± 0.02	0.88 ± 0.04	1.65 ± 0.12	1.53 ± 0.05
24	12.21 ± 0.81	13.06 ± 1.12	14.89 ± 0.78	12.03 ± 1.12	7.57 ± 0.54	9.03 ± 0.48	7.59 ± 1.08	6.32 ± 0.19	13.62 ± 0.64	13.54 ± 0.61
25	4.31 ± 0.19	6.16 ± 0.45	4.71 ± 0.73	5.71 ± 0.68	2.91 ± 0.14	2.76 ± 0.14	3.15 ± 0.19	3.35 ± 0.23	5.91 ± 0.37	6.24 ± 0.65
26	4.21 ± 0.28	5.98 ± 0.26	4.94 ± 0.17	5.25 ± 0.42	2.01 ± 0.16	2.68 ± 0.17	3.09 ± 0.19	2.27 ± 0.31	4.53 ± 0.51	5.37 ± 1.01
27	3.31 ± 0.34	3.35 ± 0.23	3.11 ± 0.07	3.42 ± 0.23	1.75 ± 0.45	2.10 ± 0.21	2.49 ± 1.81	1.85 ± 0.38	3.72 ± 0.19	4.33 ± 0.51
28	2.07 ± 0.11	2.25 ± 0.04	2.26 ± 0.16	2.71 ± 0.12	1.16 ± 0.27	2.12 ± 0.13	2.28 ± 0.67	1.53 ± 0.05	2.82 ± 0.14	3.36 ± 0.31
29	8.54 ± 0.57	10.94 ± 0.53	8.90 ± 1.01	9.97 ± 0.64	5.18 ± 0.45	5.82 ± 0.36	6.54 ± 0.38	5.29 ± 0.45	11.43 ± 0.91	11.87 ± 0.87
30	0.57 ± 0.02	0.48 ± 0.04	0.53 ± 0.03	0.49 ± 0.02	0.61 ± 0.02	0.61 ± 0.03	0.52 ± 0.02	0.72 ± 0.03	1.04 ± 0.04	0.78 ± 0.08
31	2.44 ± 0.27	2.87 ± 0.53	2.87 ± 0.32	2.76 ± 0.54	1.57 ± 0.21	1.91 ± 0.09	1.64 ± 0.12	1.57 ± 0.21	3.49 ± 0.11	3.36 ± 0.02
32	0.24 ± 0.01	0.25 ± 0.02	0.25 ± 0.02	0.27 ± 0.01	0.24 ± 0.00	0.24 ± 0.01	0.26 ± 0.01	0.23 ± 0.00	0.42 ± 0.02	0.43 ± 0.02
33	0.09 ± 0.00	0.13 ± 0.02	0.10 ± 0.01	0.14 ± 0.01	0.11 ± 0.02	0.10 ± 0.01	0.10 ± 0.01	0.09 ± 0.00	0.17 ± 0.01	0.17 ± 0.01
34	4.03 ± 0.09	4.71 ± 0.15	4.26 ± 0.13	4.62 ± 0.07	1.73 ± 0.37	2.32 ± 1.56	2.98 ± 1.34	1.94 ± 0.21	3.68 ± 1.11	3.83 ± 0.21
35	21.43 ± 2.01	16.09 ± 1.97	15.59 ± 1.12	14.89 ± 0.21	15.80 ± 0.21	8.29 ± 1.18	9.36 ± 0.45	8.81 ± 1.17	19.61 ± 1.24	20.77 ± 1.58
36	3.83 ± 1.12	4.59 ± 1.57	3.73 ± 1.16	4.29 ± 1.66	2.16 ± 0.57	2.62 ± 0.07	2.48 ± 0.08	2.15 ± 0.58	4.77 ± 1.56	4.82 ± 1.45
37	0.43 ± 0.02	0.52 ± 0.02	0.50 ± 0.01	0.46 ± 0.00	0.45 ± 0.01	0.61 ± 0.03	0.39 ± 0.02	0.39 ± 0.03	1.19 ± 0.21	0.87 ± 0.04
38	5.79 ± 0.75	8.95 ± 0.58	8.08 ± 1.01	8.87 ± 1.22	4.16 ± 0.76	5.48 ± 1.43	6.17 ± 0.17	4.22 ± 0.13	7.73 ± 0.97	8.52 ± 1.04
39	11.85 ± 0.74	14.11 ± 1.02	12.64 ± 0.35	14.51 ± 1.02	7.86 ± 0.13	9.50 ± 0.15	8.75 ± 1.62	7.39 ± 1.44	15.07 ± 1.21	15.72 ± 1.43
40	2.21 ± 0.13	2.81 ± 0.14	2.85 ± 0.53	2.98 ± 0.18	1.40 ± 0.19	1.76 ± 0.48	2.18 ± 0.17	1.68 ± 0.15	2.98 ± 0.17	3.00 ± 0.81
41	14.70 ± 0.47	17.12 ± 1.02	13.96 ± 1.02	17.05 ± 0.98	12.47 ± 2.01	8.82 ± 1.78	12.41 ± 0.45	11.25 ± 0.56	18.76 ± 1.87	20.12 ± 2.12
N ^c^	25.40 ± 3.81	41.74 ± 5.42	26.89 ± 2.83	47.29 ± 6.14	17.59 ± 2.15	19.88 ± 1.98	32.20 ± 2.21	26.71 ± 2.72	66.57 ± 4.72	60.22 ± 5.29
A ^d^	144.32 ± 15.61	182.85 ± 18.39	150.86 ± 12.70	174.24 ± 21.74	95.97 ± 8.45	91.82 ± 10.83	110.78 ± 14.92	98.16 ± 10.84	199.25 ± 17.81	208.34 ± 20.37
Total	169.71 ± 15.78	224.59 ± 22.74	177.74 ± 16.82	211.53 ± 25.62	113.56 ± 13.00	111.70 ± 7.38	142.98 ± 14.71	124.88 ± 13.80	265.81 ± 22.89	268.55 ± 27.85
**Analyte ^a^**	**Sample (mg/g, *n* = 3)**									
	**F3**	**H4**	**G4**	**S4**	**F4**	**H5**	**G5**	**S5**	**F5**	
1	0.27 ± 0.03	0.16 ± 0.02	0.15 ± 0.02	0.20 ± 0.01	0.18 ± 0.00	0.16 ± 0.01	0.15 ± 0.02	0.20 ± 0.02	0.17 ± 0.01	
2	0.07 ± 0.01	0.16 ± 0.02	0.08 ± 0.02	0.09 ± 0.01	0.10 ± 0.01	0.25 ± 0.02	0.13 ± 0.01	0.05 ± 0.00	0.06 ± 0.01	
3	0.12 ± 0.03	0.15 ± 0.01	0.10 ± 0.01	0.09 ± 0.02	0.08 ± 0.02	0.10 ± 0.00	0.33 ± 0.02	0.16 ± 0.02	0.09 ± 0.02	
4	2.15 ± 1.02	0.88 ± 0.05	1.36 ± 0.20	0.89 ± 0.08	1.60 ± 0.20	0.60 ± 0.05	0.66 ± 0.04	0.92 ± 0.05	0.85 ± 0.04	
5	1.20 ± 0.38	0.18 ± 0.03	0.61 ± 0.04	0.35 ± 0.02	0.57 ± 0.02	0.11 ± 0.01	0.15 ± 0.05	0.30 ± 0.04	0.44 ± 0.03	
6	1.92 ± 0.21	0.69 ± 0.23	1.13 ± 0.16	0.74 ± 0.02	1.39 ± 0.19	0.40 ± 0.03	0.46 ± 0.11	0.68 ± 0.04	0.53 ± 0.05	
7	0.26 ± 0.05	0.17 ± 0.04	0.18 ± 0.03	0.18 ± 0.02	0.17 ± 0.06	0.17 ± 0.03	0.17 ± 0.03	0.19 ± 0.04	0.18 ± 0.06	
8	0.22 ± 0.04	0.12 ± 0.03	0.12 ± 0.02	0.12 ± 0.01	0.12 ± 0.00	0.12 ± 0.01	0.17 ± 0.03	0.17 ± 0.04	0.16 ± 0.02	
9	0.73 ± 0.05	0.22 ± 0.04	0.22 ± 0.03	0.38 ± 0.07	0.35 ± 0.08	0.39 ± 0.09	0.44 ± 0.03	0.28 ± 0.07	0.18 ± 0.02	
10	22.54 ± 0.48	11.81 ± 0.38	20.81 ± 0.24	11.23 ± 0.45	24.70 ± 2.12	5.91 ± 0.17	8.06 ± 1.12	11.81 ± 1.32	10.39 ± 1.89	
11	2.46 ± 0.34	1.19 ± 0.03	1.76 ± 0.12	1.16 ± 0.03	2.23 ± 0.34	0.85 ± 0.02	0.81 ± 0.03	1.21 ± 0.05	1.22 ± 0.04	
12	0.19 ± 0.01	0.07 ± 0.00	0.17 ± 0.02	0.10 ± 0.01	0.11 ± 0.01	0.07 ± 0.01	0.07 ± 0.02	0.09 ± 0.03	0.13 ± 0.00	
13	0.21 ± 0.04	0.12 ± 0.01	0.09 ± 0.02	0.12 ± 0.01	0.10 ± 0.01	0.05 ± 0.00	0.52 ± 0.01	0.84 ± 0.11	0.07 ± 0.02	
14	1.23 ± 0.22	0.15 ± 0.01	0.49 ± 0.02	0.36 ± 0.03	0.12 ± 0.01	0.10 ± 0.01	0.09 ± 0.02	0.26 ± 0.05	0.18 ± 0.02	
15	2.70 ± 0.28	1.57 ± 0.51	2.55 ± 0.35	1.72 ± 0.12	2.41 ± 0.33	1.10 ± 0.03	1.44 ± 0.21	1.68 ± 0.13	1.76 ± 0.22	
16	14.41 ± 1.13	7.17 ± 1.07	10.97 ± 1.45	7.90 ± 0.65	10.61 ± 2.24	5.06 ± 0.07	6.67 ± 1.01	7.39 ± 0.23	7.85 ± 0.25	
17	61.03 ± 3.43	23.57 ± 1.75	44.71 ± 2.69	26.68 ± 2.01	32.35 ± 2.84	20.65 ± 1.05	15.25 ± 0.98	16.13 ± 1.29	21.27 ± 2.13	
18	0.03 ± 0.00	0.04 ± 0.01	0.07 ± 0.00	0.04 ± 0.01	0.03 ± 0.01	0.03 ± 0.01	0.04 ± 0.01	0.03 ± 0.01	0.04 ± 0.00	
19	4.37 ± 0.19	6.19 ± 0.92	6.25 ± 0.83	5.24 ± 0.25	5.56 ± 0.24	2.97 ± 0.17	3.89 ± 0.35	3.82 ± 0.23	3.05 ± 1.62	
20	4.45 ± 0.83	3.51 ± 0.57	5.06 ± 0.87	3.53 ± 1.17	4.02 ± 0.48	1.39 ± 0.49	2.52 ± 0.57	2.23 ± 0.18	1.91 ± 0.52	
21	0.58 ± 0.02	0.91 ± 0.11	0.99 ± 0.13	0.84 ± 0.03	0.84 ± 0.04	1.36 ± 0.26	0.50 ± 0.01	1.44 ± 0.22	1.37 ± 0.26	
22	5.57 ± 1.72	6.16 ± 1.52	8.06 ± 0.15	5.85 ± 0.18	6.83 ± 0.56	2.89 ± 0.82	4.29 ± 1.74	5.48 ± 0.32	4.17 ± 0.52	
23	1.51 ± 0.24	1.10 ± 0.04	1.97 ± 0.06	1.18 ± 0.05	1.44 ± 0.02	0.54 ± 0.03	0.81 ± 0.03	1.07 ± 0.04	0.95 ± 0.04	
24	13.72 ± 1.80	11.77 ± 0.59	13.28 ± 1.60	13.36 ± 1.80	10.20 ± 0.54	7.73 ± 0.65	9.76 ± 1.17	6.46 ± 0.54	5.98 ± 0.67	
25	5.54 ± 1.10	4.55 ± 1.04	5.75 ± 1.05	4.01 ± 0.24	4.14 ± 0.21	2.49 ± 0.14	2.82 ± 0.06	2.71 ± 0.58	3.07 ± 0.12	
26	4.40 ± 0.24	4.22 ± 0.36	6.13 ± 0.26	4.83 ± 0.19	4.03 ± 0.26	2.05 ± 0.17	2.66 ± 0.76	2.72 ± 0.86	1.99 ± 0.55	
27	2.71 ± 0.55	3.17 ± 0.15	3.43 ± 1.12	2.97 ± 0.32	2.81 ± 0.30	2.11 ± 1.01	2.19 ± 0.96	2.12 ± 0.18	1.60 ± 0.47	
28	2.16 ± 0.58	1.66 ± 0.24	1.83 ± 0.57	1.92 ± 0.57	1.69 ± 0.61	1.12 ± 0.17	1.79 ± 0.52	1.89 ± 0.57	1.28 ± 0.24	
29	10.86 ± 0.57	8.19 ± 0.35	10.75 ± 0.57	8.20 ± 0.43	7.43 ± 0.43	4.92 ± 0.81	5.89 ± 1.13	5.31 ± 0.52	4.75 ± 0.86	
30	0.78 ± 0.23	0.47 ± 0.02	0.48 ± 0.13	0.52 ± 0.21	0.47 ± 0.01	0.52 ± 0.22	0.56 ± 0.05	0.53 ± 0.04	0.67 ± 0.02	
31	2.74 ± 0.37	2.36 ± 0.48	2.82 ± 0.39	2.67 ± 0.37	2.38 ± 0.66	1.55 ± 0.21	2.03 ± 0.54	1.39 ± 0.23	1.43 ± 0.21	
32	0.39 ± 0.04	0.24 ± 0.01	0.26 ± 0.00	0.25 ± 0.02	0.27 ± 0.04	0.25 ± 0.02	0.24 ± 0.02	0.25 ± 0.03	0.25 ± 0.01	
33	0.15 ± 0.01	+ ^b^	+	+	+	+	+	+	+	
34	3.76 ± 0.32	4.50 ± 0.74	5.59 ± 1.02	4.41 ± 1.02	4.40 ± 1.12	1.79 ± 0.47	2.43 ± 0.32	3.02 ± 0.16	2.17 ± 0.38	
35	17.84 ± 1.01	20.10 ± 1.29	14.46 ± 0.92	13.68 ± 0.62	11.36 ± 0.73	17.36 ± 1.21	9.56 ± 1.02	6.62 ± 0.24	7.42 ± 1.01	
36	4.11 ± 0.76	4.32 ± 0.58	5.53 ± 0.56	3.84 ± 1.03	4.37 ± 0.63	2.17 ± 0.98	2.61 ± 0.72	2.56 ± 0.36	2.34 ± 0.46	
37	0.61 ± 0.02	0.43 ± 0.01	0.53 ± 0.01	0.44 ± 0.00	0.41 ± 0.02	0.47 ± 0.01	0.44 ± 0.02	0.34 ± 0.00	0.39 ± 0.03	
38	6.97 ± 0.26	5.22 ± 0.83	9.18 ± 1.01	7.81 ± 0.41	6.27 ± 0.78	3.05 ± 0.15	5.52 ± 0.83	5.12 ± 0.45	3.85 ± 1.01	
39	15.45 ± 1.32	16.40 ± 1.28	16.26 ± 1.37	13.70 ± 0.82	12.76 ± 1.51	8.55 ± 0.72	9.90 ± 0.50	8.02 ± 0.23	7.79 ± 0.17	
40	2.84 ± 0.22	2.23 ± 0.67	2.85 ± 0.08	2.61 ± 0.45	2.27 ± 0.98	1.28 ± 0.16	1.77 ± 0.53	1.89 ± 0.34	15.20 ± 1.28	
41	20.99 ± 0.46	15.13 ± 0.98	15.93 ± 1.39	12.30 ± 0.78	12.64 ± 1.01	12.05 ± 0.94	9.36 ± 0.27	9.16 ± 0.43	10.04 ± 0.17	
N ^c^	50.69 ± 4.81	24.80 ± 1.26	40.80 ± 4.19	25.64 ± 2.11	44.85 ± 3.62	15.43 ± 1.12	20.33 ± 2.03	26.25 ± 1.03	24.35 ± 0.78	
A ^d^	193.56 ± 18.72	146.48 ± 15.29	182.18 ± 16.50	140.91 ± 14.93	139.18 ± 11.27	99.83 ± 10.83	96.83 ± 8.23	90.35 ± 12.47	89.29 ± 11.38	
Total	244.25 ± 22.51	171.27 ± 16.78	222.98 ± 23.85	166.54 ± 15.73	184.03 ± 18.35	114.76 ± 12.46	117.16 ± 15.47	116.59 ± 14.19	113.64 ± 13.86	

The data was presented as average of three batches of samples. ^a^ Analyte number; ^b^ Below the limit of quantitation; ^c^ Total content of nucleosides and nucleobases; ^d^ Total content of amino acids.

**Table 4:** Component loading matrix of 41 amino acids, nucleosides and nucleobases for PCA.

Component (PC)	Analytes																				
	1	2	3	4	5	6	7	8	9	10	11	12	13	14	15	16	17	18	19	20	21
1	0.570	−0.374	−0.211	0.894	0.873	0.923	0.759	0.395	0.150	0.918	0.891	0.855	−0.313	0.861	0.914	0.921	0.950	0.540	0.721	0.915	0.719
2	0.695	0.146	0.236	0.376	0.384	0.310	0.574	0.813	0.242	0.106	0.319	0.347	0.215	0.446	0.257	0.296	0.156	−0.364	−0.624	−0.330	−0.660
3	−0.053	0.667	−0.017	−0.041	−0.150	−0.044	0.039	−0.121	0.494	−0.202	−0.059	−0.146	−0.605	−0.015	−0.173	−0.127	0.036	0.130	−0.003	−0.049	0.061

	22	23	24	25	26	27	28	29	30	31	32	33	34	35	36	37	38	39	40	41	
1	0.688	0.872	0.811	0.954	0.842	0.891	0.808	0.969	0.426	0.935	0.787	0.630	0.708	0.595	0.884	0.715	0.836	0.858	0.930	0.899	
2	−0.578	−0.233	−0.357	−0.199	−0.509	−0.273	0.142	−0.170	0.800	−0.173	0.556	0.448	−0.677	−0.106	−0.417	0.479	−0.357	−0.374	−0.241	0.051	
3	−0.324	−0.328	0.296	0.048	−0.031	0.105	−0.221	0.084	0.136	0.185	0.110	0.049	−0.094	0.724	0.057	0.215	−0.210	0.196	−0.115	0.266	

**Table 5:** *Sipunculus nudus* samples.

NO	Sample	Anatomical Part	Habitat	Processing Method
1	H1	Body wall (SE)	Hongkong	Ultrasonication
2	G1	Body wall (SE)	Guangxi	Ultrasonication
3	S1	Body wall (SE)	Shandong	Ultrasonication
4	F1	Body wall (SE)	Fujian	Ultrasonication
5	H2	Intestine (SI)	Hongkong	Ultrasonication
6	G2	Intestine (SI)	Guangxi	Ultrasonication
7	S2	Intestine (SI)	Shandong	Ultrasonication
8	F2	Intestine (SI)	Fujian	Ultrasonication
9	G3	Coelomic fluid (SC)	Guangxi	Ultrasonication
10	S3	Coelomic fluid (SC)	Shandong	Ultrasonication
11	F3	Coelomic fluid (SC)	Fujian	Ultrasonication
12	H4	Body wall (SE)	Hongkong	Refluxing
13	G4	Body wall (SE)	Guangxi	Refluxing
14	S4	Body wall (SE)	Shandong	Refluxing
15	F4	Body wall (SE)	Fujian	Refluxing
16	H5	Intestine (SI)	Hongkong	Refluxing
17	G5	Intestine (SI)	Guangxi	Refluxing
18	S5	Intestine (SI)	Shandong	Refluxing
19	F5	Intestine (SI)	Fujian	Refluxing

Three batches of each sample were collected from the different locations.

## Supplementary Files

Supplementary File 1
