# Supplementary material for: Simultaneous Quantitation of Free Amino Acids, Nucleosides and Nucleobases in Sipunculus nudus by Ultra-High Performance Liquid Chromatography with Triple Quadrupole Mass Spectrometry
Source: Molecules. 2016 Mar 25;21(4):408. doi: 10.3390/molecules21040408 (PMC6273726; doi:10.3390/molecules21040408)
Supplement: Supplementary File 1 [file molecules-21-00408-s001.pdf]

Supplementary Materials: Simultaneous Quantitation of Free Amino Acids, Nucleosides and Nucleobases in Sipunculus nudus by Ultra-High Performance Liquid Chromatography with Triple Quadrupole Mass Spectrometry

Yahui Ge, Yuping Tang, Sheng Guo, Xin Liu, Zhenhua Zhu, Lili Zhang, Pei Liu, Shaoxiong Ding, Xiangzhi Lin, Rurong Lin and Jin-ao Duan

To compare the determination results with and without internal standard, we selected adenosine cyclophosphate as internal standard since it does not exist in all the samples of the experiment.

A mixed standard stock solution containing the reference compounds 1–41 dried to constant weight was prepared in methanol/water (9:1, *v*/*v*). One 1 mL mixed standard solution was taken out and added deionized water in the same volume, and another 1 mL mixed standard solution was taken out and added internal standard solution the same volume, and a sample was treated in the same way. CA (the concentration of sample solution determined without internal standard) and CB (the concentration of sample solution determined with internal standard) were calculated by calibration curves established by one point external standard method. The T test method was applied to test the significance of difference between CA and CB, and the results indicated compared to CA, CB of most components except five (inosine, 2′-deoxyguanosine, guanine, glycine and lysine) did not show significant difference ($p > 0.05$). The details of T test showed in Table S1.

Table S1. T test for C_A and C_B of 41 components

NO	Component	C_A (mg/mL)	C_B (mg/mL)	P
		0.0057	0.0063	
1	Adenosine 5′-monophosphate	0.0058	0.0063	0.2988
		0.0056	0.0055	
		0.0027	0.0035	
2	Inosine	0.0028	0.0034	0.0112
		0.0024	0.0040	
		0.0050	0.0041	
3	Guanosine	0.0044	0.0043	0.1806
		0.0043	0.0043	
		0.0212	0.0178	
4	Thymidine	0.0197	0.0151	0.0640
		0.0177	0.0154	
		0.0089	0.0072	
5	2′-deoxyuridine	0.0076	0.0077	0.1613
		0.0076	0.0066	
		0.0230	0.0218	
6	2′-deoxyinosine	0.0279	0.0250	0.5885
		0.0249	0.0258	
		0.0020	0.0020	
7	Cytidine-5′-monophosphate	0.0024	0.0020	0.0732
		0.0024	0.0018	

		0.0048	0.0057	
8	2′-Deoxyadenosine-5′-monophosphate	0.0054	0.0046	0.7187
		0.0045	0.0048	
		0.0096	0.0088	
9	2′-deoxycytidine	0.0117	0.0100	0.0822
		0.0113	0.0085	
		0.3014	0.1875	
10	2′-deoxyguanosine	0.2777	0.2061	0.0040
		0.2568	0.1871	
		0.0283	0.0273	
11	Thymine	0.0263	0.0257	0.1624
		0.0312	0.0241	
		0.0032	0.0031	
12	Adenine	0.0036	0.0025	0.1175
		0.0031	0.0029	
		0.0021	0.0020	
13	Cytidine	0.0024	0.0022	0.1076
		0.0026	0.0018	
		0.0057	0.0044	
14	Uracil	0.0062	0.0050	0.1026
		0.0054	0.0054	
		0.0505	0.0328	
15	Guanine	0.0434	0.0299	0.0029
		0.0453	0.0322	
		0.3132	0.3029	
16	Xanthine	0.2988	0.2843	0.1253
		0.3088	0.2641	
		1.1131	0.8857	
17	Glycine	1.2313	0.7568	0.0197
		1.0125	0.8679	
		0.0028	0.0042	
18	GABA	0.0034	0.0035	0.0594
		0.0031	0.0037	
		0.1451	0.1036	
19	Leucine	0.1345	0.1151	0.0787
		0.1145	0.1080	
		0.1105	0.0787	
20	Isoleucine	0.1017	0.0877	0.0517
		0.0890	0.0765	
		0.0421	0.0326	
21	Methionine	0.0380	0.0373	0.0752
		0.0379	0.0303	
22	Phenylalanine	0.1123	0.0970	0.1190

		0.1235	0.1099	
		0.1067	0.0877	
		0.0377	0.0374	
23	Tryptophan	0.0434	0.0436	0.8021
		0.0379	0.0355	
		0.5245	0.4556	
24	Alanine	0.4234	0.5038	0.8022
		0.4766	0.4368	
		0.1456	0.1215	
25	Threonine	0.1320	0.1110	0.1523
		0.1277	0.1310	
		0.2566	0.1914	
26	Serine	0.2200	0.2099	0.0723
		0.2172	0.1872	
		0.1516	0.1303	
27	Asparagine	0.1365	0.1102	0.1239
		0.1270	0.1208	
		0.0842	0.0728	
28	Glutamine	0.0704	0.0658	0.2791
		0.0694	0.0654	
		0.4038	0.3484	
29	Glutamate	0.3715	0.3271	0.0888
		0.3460	0.3026	
		0.0652	0.0439	
30	Citrulline	0.0560	0.0508	0.1203
		0.0547	0.0544	
		0.1045	0.0979	
31	Proline	0.1123	0.0997	0.1493
		0.1234	0.1086	
		0.0182	0.0164	
32	Hydroxyproline	0.0214	0.0165	0.0558
		0.0189	0.0175	
		0.0134	0.0121	
33	Cysteine	0.0142	0.0111	0.0989
		0.0121	0.0121	
		0.4657	0.5131	
34	Tyrosine	0.5454	0.5245	0.5467
		0.4879	0.5090	
		1.2324	1.6566	
35	Taurine	1.3435	1.4457	0.0639
		1.3453	1.4365	
36	Valine	0.3345	0.3425	0.0732
		0.2789	0.3979	

		0.2990	0.3468	
		0.0165	0.0184	
37	Ornithine	0.0165	0.0184	0.0588
		0.0175	0.0209	
		0.2148	0.1919	
38	Aspartate	0.1820	0.1645	0.1056
		0.2083	0.1623	
		0.3778	0.2739	
39	Lysine	0.3302	0.2919	0.0236
		0.3298	0.2428	
		0.0410	0.0466	
40	Histidine	0.0450	0.0480	0.9632
		0.0493	0.0412	
		0.5702	0.4646	
41	Arginine	0.5256	0.4383	0.0766
		0.4669	0.4376	
